# Effectiveness of Digital Mental Health Interventions in the Workplace: Umbrella Review of Systematic Reviews

**DOI:** 10.2196/67785

**Published:** 2025-01-24

**Authors:** Gillian Cameron, Maurice Mulvenna, Edel Ennis, Siobhan O'Neill, Raymond Bond, David Cameron, Alex Bunting

**Affiliations:** 1 School of Computing Ulster University Belfast United Kingdom; 2 School of Psychology Ulster University Coleraine United Kingdom; 3 Inspire Belfast United Kingdom

**Keywords:** digital interventions for mental health, workplace wellbeing, stress, anxiety, depression, burnout, CBT, umbrella review, digital mental health, evaluation, psychological, databases, Pubmed, Web of Science, Medline, Cochrane Library, PRISMA

## Abstract

**Background:**

There is potential for digital mental health interventions to provide affordable, efficient, and scalable support to individuals. Digital interventions, including cognitive behavioral therapy, stress management, and mindfulness programs, have shown promise when applied in workplace settings.

**Objective:**

The aim of this study is to conduct an umbrella review of systematic reviews in order to critically evaluate, synthesize, and summarize evidence of various digital mental health interventions available within a workplace setting.

**Methods:**

A systematic search was conducted to identify systematic reviews relating to digital interventions for the workplace, using the PRISMA (Preferred Reporting Items for Systematic Reviews and Meta-Analysis). The review protocol was registered in the Open Science Framework. The following databases were searched: PubMed, Web of Science, MEDLINE, PsycINFO, and Cochrane Library. Data were extracted using a predefined extraction table. To assess the methodological quality of a study, the AMSTAR-2 tool was used to critically appraise systematic reviews of health care interventions.

**Results:**

The literature search resulted in 11,875 records, which was reduced to 14 full-text systematic literature reviews with the use of Covidence to remove duplicates and screen titles and abstracts. The 14 included reviews were published between 2014 and 2023, comprising 9 systematic reviews and 5 systematic reviews and meta-analyses. AMSTAR-2 was used to complete a quality assessment of the reviews, and the results were critically low for 7 literature reviews and low for the other 7 literature reviews. The most common types of digital intervention studied were cognitive behavioral therapy, mindfulness/meditation, and stress management followed by other self-help interventions. Effectiveness of digital interventions was found for many mental health symptoms and conditions in employee populations, such as stress, anxiety, depression, burnout, and psychological well-being. Factors such as type of technology, guidance, recruitment, tailoring, and demographics were found to impact effectiveness.

**Conclusions:**

This umbrella review aimed to critically evaluate, synthesize, and summarize evidence of various digital mental health interventions available within a workplace setting. Despite the low quality of the reviews, best practice guidelines can be derived from factors that impact the effectiveness of digital interventions in the workplace.

**Trial Registration:**

OSF Registries osf.io/rc6ds; https://doi.org/10.17605/OSF.IO/RC6DS

## Introduction

### Digital Mental Health Interventions in the Workplace

Digital mental health interventions can provide support to individuals, in a potentially low cost, efficient and scalable way. Digital interventions that have been generalized from clinical or community settings, to the workplace, primarily cognitive behavioral therapy (CBT) [1], stress management [2], and mindfulness-based stress reduction programs [3] have shown promise.

However, a criticism of digital interventions for mental health is their absence of evidence-based frameworks. A review of 293 commercially available apps for anxiety and depression found just over 55% contained information on an evidence-based framework, and of these only 6.2% published evidence to support their efficacy [4]. Furthermore, in 2020, the Organization for the Review of Care and Health Apps, a UK organization that assesses the quality of digital health apps, reviewed almost 600 mental health apps that are commercially available, and only 29.6% met their quality thresholds across different criteria including clinical assurance, data privacy, and user experience [5].

Implementation of evidence-based digital mental health interventions into real-world care is therefore lacking, resulting in the need to prioritize research exploring workflow considerations [6]. Linardon et al [7], found that studies that took a blended approach improved engagement (a blended approach includes human support supplementing a digital intervention). There is also a lack of gold-standard evidence and best practices to determine which interventions are effective for specific industry sectors or workforce populations alongside identifying those that may potentially cause harm [8].

Torous et al [6], also highlight the need to rethink and expand traditional integrated care pathways to include digital interventions to maximize their full benefit, where rapid advances in digital health technology capabilities, digital health standards, and regulation, the post–COVID-19 era presents a unique opportunity to realize this ambition. Digital interventions, used in conjunction with and to enhance traditional support options could provide a way to encourage help-seeking within an employee assistance program, and provide different industries and occupational groups with personalized well-being support.

Many systematic reviews focus on specific delivery methods of digital interventions, such as “web-based” or a “mobile app” delivered in the workplace. There are also reviews focused solely on specific mental health conditions and methods of treatment, such as stress or mindfulness. There are also existing reviews that focus on specific workplace populations, such as “white-collar” workers, health care professionals, and teachers.

### Aim

The aim of this study is to conduct an umbrella review of systematic reviews to critically evaluate, synthesize and summarize the best available evidence for a variety of digital mental health interventions currently available within a workplace setting, identifying those which enhance mental health and well-being alongside highlight gaps in the knowledge base to guide further research.

## Methods

### Overview

A systematic search was conducted in January 2024 to identify systematic reviews relating to the effectiveness of digital interventions for a workplace population. The following five databases were searched: (1) PubMed, (2) Web of Science, (3) MEDLINE, (4) Cochrane Library, and (5) PsycINFO. Three categories of search terms were used as follows: (1) “mental health,” (2) “digital interventions,” and (3) “workplace”, with the Boolean operator “AND” to separate categories and “OR” within categories. The search strategy for each database is presented in Multimedia Appendix 1. Google Scholar was also used to identify any further systematic reviews. The review protocol was registered in the Open Science Framework [9].

### Search Terms

#### Mental Health and Well-Being

The search terms were as follows: depress* OR anxiet* OR anxious OR mood OR “mental health” OR “psychological wellbeing” OR “mental wellbeing” OR “behavioral health” OR “mental illness” OR stress [10].

#### Digital Interventions

The search terms were as follows: “online intervention” OR “online treatment” OR “digital intervention” OR “digital treatment” OR “mobile intervention” OR “mobile treatment” OR “smartphone intervention” OR “smartphone treatment” OR “web-based intervention” OR “web based treatment” OR “internet intervention” OR “internet treatment” OR “computer intervention” OR “computer treatment” OR “cyber intervention” OR “cyber treatment” OR “electronic intervention” OR “electronic treatment” OR ( mobile AND program* ) OR mhealth OR ehealth OR mtherap* OR etherap* OR telehealth OR telemedicine OR “mobile app*” [10].

#### Workplaces

The search terms were as follows: workplace OR occupation* OR “work place” OR worksite OR office OR work [11].

### Study Selection and Inclusion

Covidence, a software designed to streamline the systematic review process was used to determine which studies were systematic reviews. Using keywords “systematic” and “review,” systematic reviews were identified by their title or abstract.

Systematic reviews covering the effectiveness of digital mental health interventions within a working adult population were included. Exclusion criteria were (1) studies that did not assess effectiveness or impact on mental health or well-being outcomes, (2) nonsystematic reviews, and (3) studies that were not available in English.

### Data Extraction and Quality Assessments

The following data were extracted using a predefined extraction table as follows: journal, publication year, databases searched, number of studies, types of study design, time period, population details, sample size (overall and mean if reported), intervention type, main findings, and limitations. The extracted data is presented in Multimedia Appendix 2 [1,8,12-23]. To assess the methodological quality of a study, the AMSTAR-2 tool was used to critically appraise systematic reviews of health care interventions, that include randomized and nonrandomized studies [24].

The critical domains AMSTAR-2 evaluates are having a protocol registered before the review begins, adequacy of the literature search, justification for excluding studies, risk of bias, and considering this when interpreting results, and appropriate meta-analytical methods and likely impact of publication bias if a meta-analysis was conducted. One author carried out the AMSTAR-2 assessments (GC), and a second author (EE) checked the assessment outcomes independently. Any queries and conflicts were resolved through consensus between some members of the team.

## Results

### Overview

The literature search extracted a total of 11,871 studies with a further 4 studies identified through Google Scholar. A total of 2463 duplicates were removed using Covidence, and a further 6774 were excluded as they were not systematic reviews. Reviewing titles and abstracts of 2638 reviews resulted in a further 2597 studies being excluded based on the inclusion and exclusion criteria.

**Figure 1:**
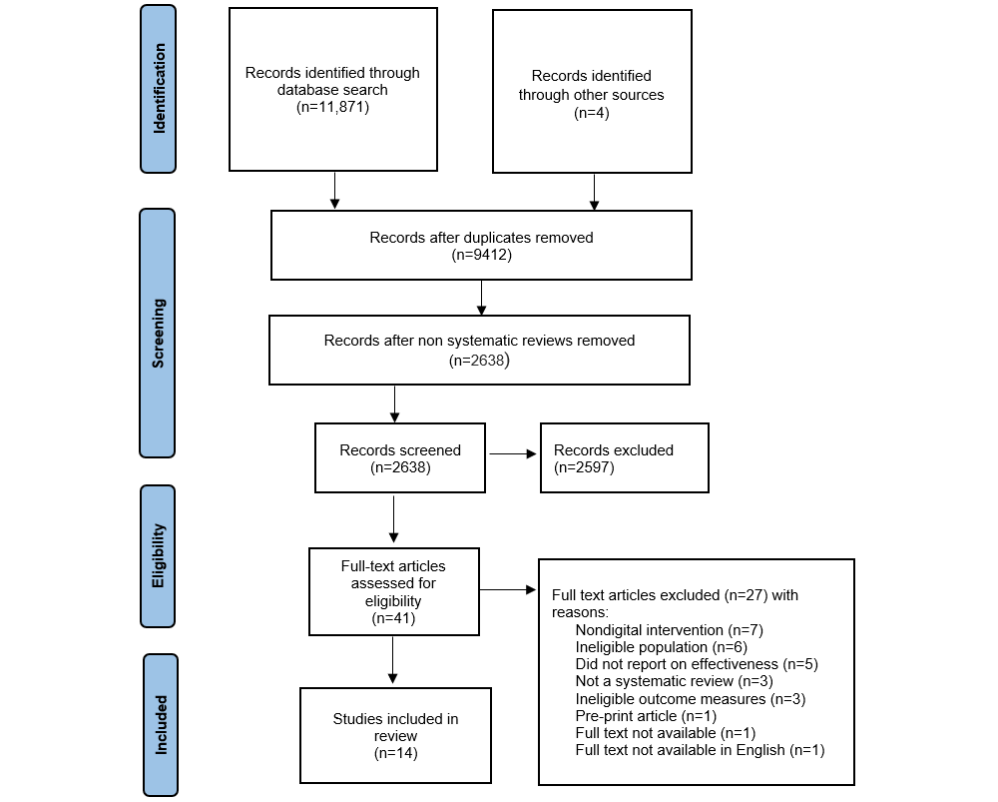
PRISMA (Preferred Reporting Items for Systematic Reviews and Meta-Analysis) flowchart showing identification, screening, and inclusion of systematic review.

A total of 41 full-text articles were then assessed for eligibility and 27 were excluded for the following reasons: (1) reviews that did not focus on digital interventions (n=7), (2) had an ineligible population—not employed individuals (n=6), (3) did not study effectiveness (n=5), (4) were not reviews (n=3), (5) did not study mental health (n=3), (6) other factors such as prepublished work (n=1), (7) full-text version not being available in English (n=1), or (8) another review’s full-text was requested, with no response (n=1). The final number of reviews included in this systematic review was 14. Figure 1 shows the PRISMA (Preferred Reporting Items for Systematic Reviews and Meta-Analysis) flowchart, and Multimedia Appendix 3 presents the PRISMA checklist.

### General Characteristics of Reviews

The 14 included reviews comprised of systematic reviews (n=9) and systematic reviews with meta-analysis (n=5). The full characteristics of this umbrella review are detailed in Table 1. The specific criteria for inclusion were notably varied across each of the reviews; most reviews only included randomized controlled trials (RCTs; n=7). Vertola et al [12] included mainly RCTs, and one clinical trial that compared 2 interventions; however, the participants were not randomized to either of the groups. Other study designs included RCTs, quasi-experimental research [13], and quantitative or qualitative research design [14].

Stratton et al [15] required studies to have a control group to meet their inclusion criteria. Narváez et al [16] did not state the specific inclusion criteria but required the methodology to be published. López-Del-Hoyo et al [17] specified that RCTs, nonrandomized trials, and single-arm studies were eligible for inclusion in the review. In their review, Drissi et al [18] examined published research that focused on digital mental health solutions for health care workers in the context of COVID-19.

**Figure 2:**
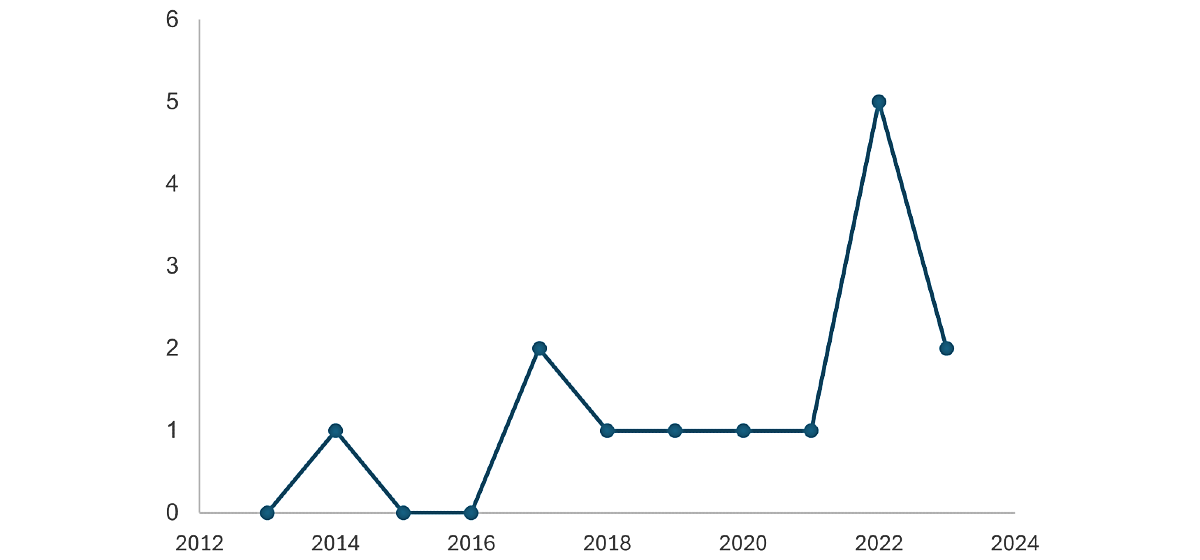
The number of systematic reviews by year.

Most systematic reviews (n=9) included a wide range of workplace sectors, including health care, manufacturing, IT, public services, and education. Two reviews did not report on the type of sector included in the studies [12,16] and 1 review included studies solely including nurses [19] and 2 on health care workers [17] with one focusing specifically on the time of COVID-19 [18]. Reviews were published from 2014 to 2023, with Figure 2 depicting the number of systematic reviews by year.

**Table 1:** Characteristics of systematic reviews and meta-analyses of digital mental health interventions for the workplace.

Author and Year	Number of studies	Population	Digital intervention context	Outcomes	Main findings	Quality checklist used
Xiong et al (2023) [20]	19	Studies were across multiple sectors including local authorities, health care, IT, and education	Internet-based cognitive behavioral therapy (iCBT) Platform used: Computer-based, web-based, mobile-based, and app-based	Depression	Computer-based, web-based, mobile-based, and app-based interventions all have potential to improve depression disorder among employees. In the RCTs^a^, iCBT demonstrated small (Hedges g=0.31, 95% CI 0.17-0.44; *P*<.001) potentially sustained effects on employees’ mental health	Cochrane risk-of-bias tool (RoB 2)
Park et al (2022) [19]	7	Study focused on the nursing staff population	Career identity training (n=1) Stress management program (n=1), Positive Thinking (n=1), Cognitive rehearsal intervention (n=1), Emotional freedom technique (n=1), Biofeedback training (n=1), and Work functioning (n=1) Platform used: web-based (n=3), smartphone-based (n=3), and real-time web-based intervention (n=1)	Burnout Secondary outcomes included workplace measures, such as career identity, workplace bullying, turnover, distress, and work They also looked at anxiety and resilience	One study reported on Burnout (primary outcome) found significantly lower levels of burnout compared with the control group who had no intervention (*P*<.001) Secondary outcomes were also reportedly improved	Cochrane’s Risk of Bias
Stratton et al (2022) [15]	75	Most studies included health care professionals (n=18), insurance industry (n=7), managers (n=6), IT (n=6), education (n=6), male-dominated industries (n=5), telecommunications (n=5), marketing and sales (n=3), banking (n=1), and HR (n=1)	Three most common interventions were based on CBT^b^, Stress management, and mindfulness. Platform used: Most interventions were delivered via web-based platforms as opposed to smartphone apps.	Depression, anxiety, and stress	Found that the body of evidence for workplace digital interventions has tripled in the past decade, but no evidence to support effectiveness has increased Found small positive effects on anxiety (Hedges g=0.26, 95% CI 0.13-0.39; *P*<.001) For depression there was small positive effects (Hedges g=0.26, 95% CI 0.19-0.34; *P*<.001) And stress (Hedges g=0.25, 95% CI 0.17-0.34; *P*<.001)	Cochrane risk of bias tool for RCTs (RoB version 2.0)
Vertola et al (2022) [12]	11	Adult workers—sectors not specified	Mindfulness/meditation, stress, well-being, mental health psychoeducation, sleep quality, and emotional regulation Platform used: mobile apps	Well-being (general and work-related) Anxiety, depression, stress, and perceived stress Job stress, emotional labor, self-regulation, life satisfaction, compassion satisfaction, and burnout	Range of outcomes, studies reported an increase in well-being (n=7), reduction in perceived stress (n=4), reduction in stress (n=3), decrease in anxiety symptoms (n=2), decrease in depressive symptoms (n=2), reduction in burnout symptoms (n=2), and decrease in job stress (n=2)	Not reported (NR)
Armaou et al (2022) [13]	51	RCTs were across multiple sectors, with the most in health care (n=8), technology/IT companies (n=5), manufacturing (n=3), quasi-experimental studies were across multiple sectors, most within health care (n=11), governmental or public enterprises (n=3), and university employees (n=2)	Categorized into 4 clusters of interventions, “self-help interventions” (n=18), followed by Stress management (n=14), Mindfulness/meditation (n=14), and CBT (n=5) Platform used: A mix of web-based and smartphone-based interventions	Primary Outcomes grouped into 3 areas: Mental Health concerns: Depression, anxiety, and dysfunctional attitudes. Work-related well-being: Perceived stress, psychological distress, and job strain Psychological wellness: general mental well-being/positive mental health, happiness, and life satisfaction. Mindfulness and resilience, self-efficacy, coping, and gratitude	Mental Health concerns: 10/51 (19.6%) Studies reported positive effects Work-related well-being: 28/51 (54.9%) Studies reported positive effects Psychological wellness indicators: 19/51 (37.3%) studies reported positive effects	Cochrane Collaboration’s Risk of Bias and JBI Critical Appraisal Checklist
Moe-Byrne et al (2022) [21]	7	Participants were recruited from a variety of workplaces. Office-based organizations (n=4), health care professionals (n=2), and private and public sector (n=1)	All studies used CBT as a theoretical background: 2 stated the use of mindfulness and 2 used stress model or the job demands resources model Increase well-being: preventative interventions (n=3) and work performance and occupational health guidance (n=1) Platform used: A web-based intervention (n=5), a smartphone app intervention (n=1), and a combined web-based and smartphone app intervention (n=1)	Depression, anxiety, and stress and work-related outcomes (absenteeism, presenteeism) Physical measures: sleeping problems (n=2), sleep and workplace performance (n=1), somatization (n=1), and physical health impairment (n=1)	All studies reported psychological outcomes Significant improvement for both anxiety and depression (n=2) Significantly lower stress scores (n=3) Well-being (mixed results): significantly more well-being over time (n=1) and did not find a statistically significant positive effect (n=1) Other mental health outcomes: significant effects for positive mental health (*F*=3.46; *P*=.03; Cohen d of 0.37 at 3 months follow-up and Cohen d of 0.28 at 6 months follow-up; n=2) Employee’s worry and quality of life regarding mental health (*P*<.001 at 6 months; n=1)	Cochrane Risk of Bias tool
Drissi et al (2021) [18]	11	Health care workers	Peer support (n=2), e-learning (n=3), web-based resources (n=3), posttraumatic stress disorder (PTSD) coach (n=1), headspace (n=1), hotline (n=1), screening (n=1), and online support groups (n=1) Platform used: social media (n=2), online support platform/resources (n=8), and mobile apps (n=1)	Only 3 studies included an evaluation, 71% of participants stated one platform helped them adjust faster to the situation, and another platform was used by 82% of participants in their work or home lives, another handbook was reported to have positive qualitative feedback	Lack of empirical evidence for health care workers, evidence mainly targeted health care workers in China	NR
Paganin and Simbula (2020) [14]	31	General workers (n=11), health and social care (n=8), office-based (n=5), technology (n=2), middle managers (n=1), construction (n=1), airplane pilots (n=1), faculty members (n=1), and general workers with serious mental illness (n=1)	Behavioral change techniques (n=5), mindfulness (n=3), stress models (n=3), and CBT (n=2) Other interventions/models such as acceptance and commitment therapy did not report on the theory used (n=12) Platform used: Smartphone-based interventions	Stress management, psychological well-being, secondary outcomes of resilience, and burnout	Studies reported on positive results for well-being and stress management—on intervention effectiveness, usability, and feasibility	NR
Phillips et al (2019) [1]	50	Varied sectors: IT (n=7), health care (n=6), education (n=3), communication and media (n=3), public sector (n=3), and banking (n=2)	CBT (n=22) Personalized feedback: general health check (n=7), mindfulness (n=6), psychoeducation (n=5) Remaining studies used a variety of training methods, such as cognitive, positive psychology, or problem-solving Platform used: web-based interventions (n=47) and smartphone- or app-based interventions (n=3)	Stress, depression, anxiety, burnout, insomnia, mental well-being, mindfulness, and alcohol intake	22 studies on stress had a medium positive effect on perceived stress (with g=0.54 (95% CI 0.35-0.72, *P*<.001) 17 studies with depression as an outcome observed a significant small positive effect (g=0.30, 95% CI 0.18-0.42, *P*<.001) And 15 studies on anxiety had a small positive effect on anxiety (g=0.34; 95% CI 0.18-0.50, *P*=.0001)	Cochrane risk-of-bias tool (RoB 2)
Howarth et al (2018) [22]	22	Variety of workplaces: public and private companies, health care professionals, and education and manufacturing plants	Interventions aimed at improving alcohol (n=5); mental health (n=5); sedentary behavior (n=3); musculoskeletal symptoms (n=2); heart health (n=2); insomnia (n=1); mix of work-related rumination, fatigue, and sleep (n=1); and mix of outcomes including coping, diet, stress, and general health (n=3) Platform used: web-based (n=11), web-based with email (n=5), web-based with both email and SMS (n=2), downloaded software (n=2), web-based with SMS (n=1), and smartphone with SMS (n=1)	Psychological measures: anxiety and depression (n=6). Others include mindfulness and help-seeking attitude Workplace measures: job stress, work engagement, and work productivity	Studies reported positive significant (N=9) findings for sedentary behavior (n=3); mental health (n=2); job satisfaction (n=1); diet, exercise, self-efficacy (n=1); insomnia (n=1); and work-related levels of rumination, problem-solving, pondering, fatigue, and sleep quality (n=1)	Cochrane’s Risk of Bias
Stratton et al (2017) [8]	23	Studies were across multiple sectors including education, health care, manufacturing, IT, and media	CBT intervention (n=11), stress management (n=6), and mindfulness-based approaches (n=6) Platform used: mixed Web-based (n=20) and smartphone (n=3)	Effectiveness: stress, anxiety, and depression	Overall, postintervention found a significant small effect (g=0.24, 95% CI 0.13 to 0.35, *P*≤.001) For CBT, a significant but very small positive effect was found Mindfulness had a moderate to large effect, but stress management interventions produced a nonsignificant small positive effect	Downs and Black checklist Risk of Bias using the Cochrane Guidelines
Carolan et al (2017) [23]	21	Most studies were from the general working population (n=4), local authorities (n=3), education (n=3), and technology (n=2)	CBT-based (n=12), stress and coping (n=3), mindfulness (n=2), social cognitive theory (n=1), positive psychology (n=1), problem-solving training (n=1), acceptance and commitment therapy (n=1), self-guided (n=11), and some guidance (n=10) Platform used: web-based (n=17), computer app (n=2), email (n=1), and standalone computer (n=1)	Psychological well-being and work effectiveness	Found digital interventions had statistically significant positive effects on psychological well-being (g=0.37, 95% CI 0.23-0.50) and work effectiveness (g=0.25, 95% CI 0.09-0.41) when compared with the control group	Cochrane Collaboration’s risk of bias tool
Narváez et al (2014) [16]	21	NR	CBT (n=10), combination of therapies (n=5) problem-solving therapy (n=1), and other types of therapies (n=5) Platform used: web-based (n=17), sensor networks (n=2), and mobile (n=1)	Occupational stress	12 studies had a positive effect on occupational stress, 3 had a positive effect but were not statistically significant, 2 studies had an indefinite effect	NR
López-Del-Hoyo et al (2023) [17]	27	Health care workers	22 interventions emerged, the authors classified digital interventions into their format “self-guided versus guided”, and their contents “third wave” psychotherapies which described mindfulness interventions versus others 18/22 interventions were self-guided, 14 of which were web-based, 3 smartphone apps, and one based on text messages	Primary outcome: stress, depressive symptoms, anxiety, burnout, resilience, and mindfulness	13 interventions produced significant posttreatment reductions in stress levels; there were also significant improvements found for depressive symptoms, anxiety, burnout, resilience, and mindfulness	Heart, Lung, and Blood Institute assessment tools

^a^RCT: randomized controlled trial.

^b^CBT: cognitive behavioral therapy.

### Quality Assessment of Reviews

AMSTAR-2 was used to complete a quality assessment of the reviews included in this umbrella review, the results were Critically Low (n=7), and Low (n=7).

A total of 9 studies provided reasons for excluding articles, however, none of the reviews provided a full list of studies that were excluded. Another noncritical item that no review met was item 10, reporting on the funding of primary studies. A total of 11 studies declared no conflict of interest or provided justification, and only one study met the full “adequacy of the literature search” critical item. Many systematic reviews (n=11) did include the components of PICO (Population, Intervention, Comparison, and Outcomes). Although many reviews (n=9) provided an overarching list of reasons why full-text articles were excluded, none of the reviews provided a full list detailing all potentially relevant studies that were read and excluded. The judgment for each AMSTAR-2 critical item area for each systematic review can be found in Multimedia Appendix 4 [1,8,12-23].

In terms of the risk of bias checklists used within the individual systematic reviews, Cochrane risk of bias (n=9) was the most widely used, followed by JBI (Joanna Briggs Institute) Critical Appraisal Checklist (n=1), Downs and Black Checklist (n=1) and Heart, Lung, and Blood Institute assessment tools (n=1). Armaou et al [13] used both the Cochrane and JBI Checklists, and 4 studies did not report on using any risk of bias checklists.

### Digital Interventions

#### Intervention Delivery

The type of digital platforms that were used to deliver the intervention was mostly mixed, including reviews reporting on studies that used web-based and mobile/smartphone-based interventions (n=11). Two reviews reported only on mobile/smartphone-based interventions [12,14] and one focused only on web-based interventions [23].

#### Intervention Type

The umbrella review identified 4 different types of digital interventions: CBT, mindfulness/meditation, stress management, and other self-help interventions.

This is consistent with other systematic reviews, as 2 reviews found the most common type of interventions were CBT-based, followed by stress management and mindfulness-based approaches [8,13]. Likewise, Phillips et al [1], Narváez et al [16], and Moe-Byrne et al [21] found that CBT was the most common intervention in the majority of studies they reviewed. Xiong et al [20] focused their review solely on internet-based cognitive behavioral therapy (iCBT), delivered through computer-based, web-based, mobile-based, and app-based platforms. Park et al [19] identified different, unique types of digital interventions compared with the rest of the reviews, including studies on career identity training (n=1) and biofeedback training (n=1). Drissi et al [18] reviewed studies on interventions using social media (n=2) for health care workers during the pandemic. López-Del-Hoyo et al [17] classified mindfulness interventions as “third wave” psychotherapies, versus “other” interventions. Across all reviews, the most common and frequent interventions included were CBT-based, followed by Mindfulness and stress management interventions.

### Outcome Measures

For the majority of the reviews, the primary outcome measure focused on the symptoms of Depression (n=9), this includes Xiong et al [20] who only included studies that focused on depression. Furthermore, reviews reported on studies measuring Anxiety (n=9), stress (n=5), well-being (n=5), burnout (n=5), occupational/job stress (n=4), perceived stress (n=2). Other reviews reported on outcomes such as resilience (n=2), sleep problems (n=2), mindfulness (n=2), alcohol, physical health, and help-seeking attitudes.

Park et al [19] found other workplace-related outcome measures within their review of studies within the nursing population, such as workplace bullying, career identity, quality of work life, and turnover. The review by Carolan et al [23] reported on work effectiveness, 2 on presenteeism and absenteeism. Outcome measures varied greatly across reviews, with 174 specific measures being mentioned across 9 reviews that reported on outcome measures. The most frequently used outcome measures are displayed in Table 2.

**Table 2:** Top 10 frequently used outcome measures.

Outcome Measure	Count, n
Center for Epidemiologic Studies Depression Scale (CES-D)	14
Depression Anxiety and Stress Scale 21 Items (DASS-21)	12
Perceived Stress Scale 10 Items (PSS-10)	11
Patient Health Questionnaire 9 Items (PHQ-9)	9
Insomnia Severity Index (ISI)	7
Connor-Davidson Resilience Scale (CD-RISC)	7
Kessler Psychological Distress Scale (K6)	6
Maslach Burnout Inventory (MBI)	6
Beck’s Depression Inventory (BDI)	5
The Symptoms of Distress Scale (SDS)	5

The most frequently used scales measured perceived stress, depression, anxiety, stress, insomnia, burnout, and psychological distress, however, there were 2 scales that measured workplace-specific outcomes, the Utrecht Work Engagement Scale followed by the brief job stress questionnaire. Cronbach α coefficients ranged between 0.70 and 0.95 each of the 10 most frequently used scales.

In an analysis of co-associations among outcome measures, patterns emerge regarding the use of instruments used together. The Generalized Anxiety Disorder-7 Scale for generalized anxiety was used in conjunction with the Patient Health Questionnaire-9 for depression, in a total of 4 studies. Another pair of outcome measures were the Maslach Burnout Inventory–Emotional Exhaustion (MBI-EE) for burnout, and the 10-item Perceived Stress Scale (PSS-10) for perceived stress appearing together in 3 studies. Similarly, the PSS-10 for perceived stress was paired with the Center for Epidemiologic Studies Depression Scale (CES-D) for depression in 3 studies. In terms of workplace-specific outcome measures, the Utrecht Work Engagement Scale was used in conjunction with Hospital Anxiety and Depression Scale–Anxiety, CES-D, PSS-10, Insomnia Severity Index, and MBI-EE in 2 studies.

### Effectiveness

#### CBT

Xiong et al [20] found iCBT had a small positive effect on symptoms of depression. Armaou et al [13] found 3 CBT digital interventions that had a significant effect on anxiety and depression symptoms. Stratton et al [8] found CBT-based interventions had a small significant positive effect (g=0.15, 95% CI 0.02 to 0.29, *P*=.03, I2=1.9%) on reducing mental health conditions in employees. However, López-Del-Hoyo et al [17] found that CBT-based programs did not produce significant effects on stress in health care professionals.

#### Mindfulness-Based Interventions

Positive effects for mindfulness-based interventions were found for anxiety and depression symptoms, with Stratton et al [8] finding a moderate to large positive effect size (Hedges *g*=0.60, 95% CI 0.34 to 0.85, *P*≤.001, I2=0.0%). Stratton et al [15] found mindfulness-based interventions to have moderate but higher effect sizes (Hedges *g*=0.46, 95% CI 0.28-0.64; *P*<.001) on depressive symptoms than CBT (Hedges *g*=0.11, 95% CI 0.06-0.17; *P*<.001). Vertola et al [12] reported that mobile health interventions, particularly those focused on mindfulness/meditation had moderate to high efficacy for supporting stress management and emotional self-regulation in the workplace. López-Del-Hoyo et al [17] reported a mindfulness decompression and psychoeducation program produced significant reductions in depressive and anxiety symptoms in health care professionals, however, this study did not have a control arm.

### Stress Management

Armaou et al [13] found positive effects of stress management interventions on work-related and psychological well-being, but equally found 4 studies that had no significant effect. Stratton et al [15] found stress management interventions (Hedges *g*=0.61, 95% CI 0.47-0.75; *P*<.001) to be more effective than CBT (Hedges *g*=0.11, 95% CI 0.06-0.17; *P*<.001), for stress, depression, and anxiety.

### Other Self-Help Interventions

Three mobile-based resilience interventions had no effect on resilience and 3 web-based positive psychology interventions had no effect on psychological well-being [13]. In contrast, the review of Phillips et al [1] indicated that positive psychology was more effective than CBT evidencing a larger effect size.

### Factors Influencing Effectiveness

Of the 14 reviews, there were several reported factors that potentially influenced within study effectiveness. These include the type of technology or platform used, if the intervention was guided or self-guided, recruitment of participants, tailoring or personalization of the intervention, and demographics such as gender or age.

Xiong et al [20] found that effectiveness was not impacted substantially by the type of platform iCBT was delivered on, that is, web-based or mobile-based. Park et al [19] found that studies using interventions that were mobile-based reported significant improvements compared with web-based interventions; however, their findings were not conclusive, as the studies within the review had poor methodological quality, a wide variation of interventions, and high heterogeneity of outcomes.

Carolan et al [23] did not find a significant difference between interventions that were guided (guidance by a person) or self-guided, in contrast, Phillips et al [1] found an advantage for guidance, however, their subgroup analyses were underpowered and must be interpreted with caution. Supported-guided interventions for stress and depression were found to have a larger effect size than self-guided, in contrast, there was no significant difference between guided and self-guided for anxiety [15]. In the context of health care professionals, guided interventions were limited in number, and did not sustain significant effects on stress at follow-up [17].

Howarth et al [22] found that studies targeting one specific outcome, rather than multifactorial, were more effective. Furthermore, Armaou et al [13] found that studies demonstrated a high risk of contamination effects and attrition bias, and concluded further high-quality evidence is needed. In terms of recruitment, Phillips et al [1] found that participants recruited from the community, evidenced a significant increase in the treatment effect (g=0.79) compared to those recruited within the workplace and called for future research to examine and focus on the impact of this.

Tailoring or personalizing interventions was found to be effective for presenteeism, stress and sleep, but less so for anxiety, depression, and absenteeism [21]. Furthermore, Stratton et al [15] argue that the limited evidence in the literature for tailored digital interventions suggest no greater efficacy for bespoke or tailored interventions in addressing mental health in the workplace.

A total of 8 studies reported the mean age and gender balance of some or all studies but did not comment further. Phillips et al [1] assessed age and gender as moderators for effect size, with a total population of 15,258. Older participants showed significantly higher effect sizes for stress, depression and burnout, while gender did not show any significant moderating effects.

## Discussion

### Principal Findings

This umbrella review aimed to critically evaluate, synthesize, and summarize the best available evidence of various digital mental health interventions being deployed within a workplace setting to (1) identify which digital mental health interventions are most effective for enhancing mental health and well-being in the workplace and (2) identify gaps in the knowledge base which require further research. Based on the 14 systematic reviews and meta-analyses reviewed there is evidence to support the effectiveness of digital interventions within a workplace setting.

The most common type of digital intervention was CBT, followed by mindfulness and stress management, and other more generic interventions, for example, resilience based or positive psychology interventions. Given the variation of language used to describe mental health digital interventions both in the general populations, and in the workplace, clear and concise operational definitions could help with standardization.

Web-based was the most common delivery platform, and Xiong et al [20] found evidence to suggest that type of platform did not significantly impact effectiveness. The latter indicates further consideration should be given to implementing other rapidly advancing platform technologies such as chatbots, virtual and augmented reality interventions.

In terms of mental health outcomes, the reviews found statistically significant positive effects primarily with self-report outcome measures which screen for depression, stress, anxiety, psychological well-being, and burnout. Armaou et al [13] found that digital interventions that were theory-informed were associated with increased effectiveness, in contrast, psychoeducation alone was the least effective and only minimally effective for improving well-being in the workplace. Xiong et al [20] and Stratton et al [8] found internet-based CBT was moderately effective in reducing the symptoms of depression in employees, while Xiong et al [20] called for further high-quality studies to add to this evidence base.

There was an especially high heterogeneity of outcomes measured with the assessments and tools used to measure these across many studies and within reviews. The most frequent assessment measures used were self-report screening questionnaires for stress, anxiety, and depression with moderate to high levels of reliability, concurrent and predictive validity. Studies would benefit from using well-validated, standardized mental health outcome measures to enable comparisons across studies and across populations, albeit reporting effect sizes does allow for comparing relative efficacy. Consideration should be given to how the different outcome measures used in previous studies can be harmonized for meta-analysis to enable reliable benchmarking, through using tools such as Harmony [25]. Furthermore, given the high heterogeneity of outcome measures used, a recommendation for standards bodies such as the Employee Assistance Professionals Association is to create an industry-wide standard approach to measuring mental health outcomes of employees, for example, with the Workplace Outcome Suite [26].

Very few reviews included studies that reported absenteeism or presenteeism, which are associated with anxiety and depression in the workplace [27]. Moe-Byrne et al [21] found the definition of presenteeism can vary and was not comparable between studies, as many used different assessment tools. However, a study that assessed presenteeism using a valid and reliable measure, treatment inventory of costs in patients with psychiatric disorders, found a statistically significant improvement, in contrast 2 studies using a direct measure of absenteeism found no significant improvement.

When interpreting the results of systematic reviews, the risk of bias needs to be considered. Using the AMSTAR-2 quality impact assessment for systematic reviews of RCTs or nonrandomized trials, 7 of the 14 systematic reviews were found to be low, and 7 were found to be critically low which places considerable limitations on the overall findings.

For all employees within the workplace, while considering generic well-being, there is evidence that mindfulness-based interventions may have a stronger, albeit moderate effect on employees, while stress management interventions may have a stronger effect on those employees presenting with higher levels of stress [8]. However, there is evidence that mindfulness may cause adverse effects in some populations who have been exposed to or witnessed workplace trauma, such as re-experiencing traumatic memories [28]. Employers, therefore, could consider including Trauma-Informed Mindfulness-Based Stress Reduction [29] which has shown promising results for those who have experienced interpersonal violence. There is also a lack of best practices and evidence to determine which interventions are effective for specific workforce populations alongside identifying those that may potentially cause harm [8].

Phillips et al [1] highlighted how community recruitment, as opposed to recruiting from a workplace population, improved the treatment effect. Equity in digital mental health is a key and growing concern, and often interventions are developed to reach mass populations, with no consideration given to how demographic factors such as age or gender and or mental health not least trauma-related presentations may moderate effect size or even lead to negative outcomes. Phillips et al [1] found older age was a positive moderating factor on effect size for a digital intervention targeting stress, depression, and burnout, however many developers of digital interventions do not consider digital divides, in terms of access, literacy, and skills [30]. Two reviews focused specifically on interventions for health care workers Park et al [19] and Drissi et al [18] during the time of the COVID-19 pandemic. Both reviews highlighted the lack of high-quality studies in this specific workforce population, and the poor methodological quality of studies to date. The prevalence of mental health disorders and levels of help-seeking vary among employees within different occupational groups and industry sectors, combined with a lack of robust evidence and best practices to determine which interventions are effective for specific industry sectors [8] highlighting an urgent need for future research in this area.

One review focused on studies that tailored or personalized interventions, including recommending content based on user screening, and material being tailored based on user characteristics and use data. Tailoring or personalization of digital tools to suit the needs of different populations is still uncommon but could have a significant impact [30]. Future research therefore should consider personalizing or tailoring their interventions which should include the preference of the service user while evaluating the effectiveness within different industry sectors and occupational groups.

The use of artificial intelligence (AI) and machine learning (ML) within digital mental health may have the potential to make interventions more personalized, and proactive. For example, recommender systems typically rely on AI to process large amounts of personal data these digital mental health interventions generate, to provide users with relevant services or information. AI and ML have also been applied in early detection of mental health [31] by analyzing large amounts of data, to detect early signs of mental health issues. This has the potential to then trigger the automated deployment of just-in-time interventions [32] or ecological momentary interventions [33] delivering the right support to an employee at the right time.

Key ethical considerations have been highlighted with using AI and ML in mental health [34] including lack of explainability, data privacy, ensuring users have control over their data, and understanding how it is used. Data privacy is particularly important in the context of employee’s using digital mental health interventions within the workplace, as employees need to understand if data are shared with management or health insurers [35].

Previous studies have shown the positive impact of guidance or human support supplementing a digital intervention [7] whereas the review of stress reduction interventions by Phillips et al [1] found the treatment effect was significantly increased by guidance. However, in contrast, the review by Carolan et al [23] found no significant difference between interventions that had guidance and those that did not. Torous et al [30] have identified the pressing research need to assess the “optimal degree and mode of human support necessary” to make digital interventions more effective.

Many reviews did not report on factors increasing engagement, user experience, or preference where the primary aim was evaluating effectiveness. However, the review by Carolan et al [23] suggests interventions that are shorter (6 to 7 weeks), and use notifications and tailoring to the user, may increase engagement with the intervention. Vertola et al [12] reiterate that studies that used reminder notifications such as push notifications or emails, led to improved adherence to digital interventions. Furthermore, Vertola et al [12] suggest that a short demonstration or tutorial before the first use would improve the user experience and promote ease of use and acceptance.

Although quality assurance is not included in the scope of this review, it is important to note that when implementing digital interventions employers should be aware of best practices and standards such as Organization for the Review of Care and Health Apps. Furthermore, this review looked at effectiveness rather than cost-effectiveness, and future studies should consider return on investment as an outcome, due to the large economic impact mental health in the workplace can have. Table 3 summarizes the key findings, and details recommendations for both policy and practice and future research.

**Table 3:** Key findings and recommendations.

Key findings	Recommendations
Personalization of digital interventions was found to be effective in improving employee mental health and well-being.	Consider personalizing and adapting digital interventions to suit the needs and preferences of different populations and industry sectors.
There is no consensus on the differential impact of the mix of human and digital on the effectiveness of an intervention.	Further research needs to be undertaken to understand the right blend of digital and human engagement for mental health in the workplace.
Mindfulness is the most effective generic well-being intervention for employees, and psychoeducation alone is the least effective.	Consider incorporating mindfulness-based interventions into employee well-being offerings, including trauma-informed mindfulness for specific occupational groups.
There is high heterogeneity in outcome measures used, with 108 different scales documented within the reviews.	Consider the outcome measures that were used in previous similar studies. Otherwise, consider how the different outcome measures used in previous studies can be harmonized for meta-analysis and benchmarking.
The effectiveness of the intervention is the same regardless of the platform used, whether web-based or mobile apps.	Consider all types and technologies of digital platforms to maximize reach.
There is a lack of robust evidence and best practices to determine which interventions are effective for specific industry sectors.	Robust research, such as randomized control trials need to be undertaken to clearly understand the efficacy of digital mental health interventions across different workplace populations.

### Conclusion

This umbrella review aimed to critically evaluate, synthesize, and summarize evidence of various digital mental health interventions available within a workplace setting. Broadly speaking the most common types of digital intervention being used in the workplace can be categorized as CBT-Based, Mindfulness, Stress management interventions, and other self-help interventions. The demand for talking therapies has more than doubled over the past decade reaching an all-time high of 1.83 million referrals in 23/24 [36], and where access has become a key public health concern. To help meet the increased demand the National Health Service is exploring the use of National Institute for Health and Care Excellence recommended “digitally enabled” evidence-based therapies for depression, anxiety, and trauma-related disorders potentially freeing up thousands of resource-intensive therapist hours [37].

This review provides tentative evidence that digitally enabled therapies could be usefully deployed in a workplace setting to help address work-related stress, depression, or anxiety, which, in 2022 and 2023 was the 5th most common reason given for sickness absence, accounting for an estimated 7.9% of all absences, equating to 17.1 million working days lost and costing UK employers an estimated £42 billion (US $52.6 billion) to £45 billion (US $56.4 billion) annually [38].

Digital interventions were found to moderately reduce the symptoms of stress, anxiety, depression, and burnout and increase psychological well-being. Interventions based on theory, and recruitment within the community increased effectiveness. Studies vary greatly in the outcomes they report on, and the tools used to measure these. The most effective type of intervention for generic well-being across all employee populations was mindfulness-based interventions.

Personalization or tailoring of digital interventions was found to be an effective way to improve employee well-being, and future research into this area could have a significant impact. The review found mixed and conflicting evidence on the role and impact of self-guided compared with guided help for increasing effectiveness. Further high-quality research that systematically investigates these issues in more detail could significantly add to the growing evidence base and inform best practice guidelines for developing and implementing effective digital interventions in the workplace.

### Strengths and Limitations

To the best of our knowledge, this umbrella review provides the first systematic synthesis of systematic reviews on digital interventions for mental health in the workplace. The review has several strengths as well as limitations.

The rigor, robustness, and strength of the findings depend on the quality of the studies that were included in the initial systematic reviews and meta-analysis where, using the AMSTAR-2 quality assessment, 7 of the 14 reviews were found to be low, and 7 critically low. The latter was largely attributable to one critical item which required authors to list all potentially relevant studies that were read in full-text form but excluded from the systematic review along with a justification for why they were excluded. This one critical item dropped the overall quality rating of 5 reviews from moderate to low, and one from high to low, introducing the significant risk of inherent bias. Furthermore, because many of the reviews focused broadly on general workplace populations it is possible individual studies were repeated within all the numerous reviews considered.

Finally, the heterogeneity of the various and numerous outcome measures is problematic, while it is also unclear to what extent the largely small to moderate, statistically significant effect sizes translate into clinically meaningful change—improved functioning. For the above reasons it is therefore important to exercise caution when interpreting the results.

Despite the above limitations, the umbrella review including only systematic reviews provides a useful summary and synthesis of the best available evidence on mental health workplace digital interventions for a large population of employees. In addition, extracting and synthesizing the reviews on different populations and occupational groups for example health care professionals and general workforce sectors will help fine-tune and adapt interventions for specific groups alongside identifying gaps in the research knowledge base to guide areas for further systematic exploration.

## Appendix

Multimedia Appendix 1 Search strategy.

Multimedia Appendix 2 Characteristics of reviews.

Multimedia Appendix 3 PRISMA (Preferred Reporting Items for Systematic Reviews and Meta-Analyses) checklists.

Multimedia Appendix 4 AMSTAR-2 Assessment Results.

Multimedia Appendix 5 JBI Umbrella Review Checklist.

## Abbreviations

AI: artificial intelligence
CBT: cognitive behavioral therapy
CES-D: Center for Epidemiologic Studies Depression Scale
iCBT: internet-based cognitive behavioral therapy
JBI: Joanna Briggs Institute
MBI-EE: Maslach Burnout Inventory–Emotional Exhaustion
ML: machine learning
PRISMA: Preferred Reporting Items for Systematic Reviews and Meta-Analysis
PSS-10: 10-item Perceived Stress Scale
RCT: randomized controlled trial
