# Supplementary material for: Effectiveness of Digital Mental Health Interventions in the Workplace: Umbrella Review of Systematic Reviews
Source: JMIR Ment Health. 2025 Jan 24;12:e67785. doi: 10.2196/67785 (PMC11806266; doi:10.2196/67785)
Supplement: Multimedia Appendix 1 [file mental_v12i1e67785_app1.docx]

**MEDLINE search strategy (adapted for other databases)**

noft(depress* OR anxiet* OR anxious OR mood OR “mental health” OR “psychological wellbeing” OR “mental wellbeing” OR “behavioral health” OR “mental illness” OR stress )

AND

noft(“online intervention” OR “online treatment” OR “digital intervention” OR “digital treatment” OR “mobile intervention” OR “mobile treatment” OR “smartphone intervention” OR “smartphone treatment” OR “web-based intervention” OR “web based treatment” OR “internet intervention” OR “internet treatment” OR “computer intervention” OR “computer treatment” OR “cyber intervention” OR “cyber treatment” OR “electronic intervention” OR “electronic treatment” OR ( mobile AND program* ) OR mhealth OR ehealth OR mtherap* OR etherap* OR telehealth OR telemedicine OR “mobile app*” )

AND

noft(workplace OR occupation* OR "work place" OR worksite OR office OR work)
