# Supplementary material for: Effectiveness of Digital Mental Health Interventions in the Workplace: Umbrella Review of Systematic Reviews
Source: JMIR Ment Health. 2025 Jan 24;12:e67785. doi: 10.2196/67785 (PMC11806266; doi:10.2196/67785)
Supplement: Multimedia Appendix 2 [file mental_v12i1e67785_app2.docx]

**Characteristics of systematic reviews and meta-analyses of digital mental health interventions for the workplace**

| **Authors and Year** | **Type of Review** | **No of Database & date range** | **No of studies included** | **Types of studies** | **Population** | **Digital Intervention Context** | **Outcomes** | **Main findings** | **Quality Checklist used** |
| --- | --- | --- | --- | --- | --- | --- | --- | --- | --- |
| Xiong et al. 2023 | Systematic Review and Meta-analysis | 5  Database inception to May 2021 | 19 | RCTs | Studies were across multiple sectors - including local authorities, healthcare, IT, Education | iCBT  Platform used - Computer-based, web-based, mobile-based and app-based | Depression | Computer-based, web-based, mobile-based and app-based interventions all have potential in improving depression disorder among employees. In the RCTs,  ICBT demonstrated small (Hedges’ g = 0.31, 95% CI [0.17, 0.44]; p < 0.001) potentially sustained effects on employees’ mental health | Cochrane risk of-bias tool (RoB 2) |
| Park et al. 2022 | Systematic Review | 6  Database inception until January 2021 | 7 | RCTs | Study focused on the nursing staff population | Career identity training (n = 1)  Stress management programme (n = 1), Positive Thinking (n = 1), Cognitive rehearsal intervention (n = 1), Emotional freedom technique (n = 1), Biofeedback training (n = 1) , Work functioning (n = 1)  Platform used - web-based (n = 3), smart-phone based (n=3) and real-time online intervention (n = 1) | Burnout was the primary outcome  Secondary outcomes included workplace measures, such as career identity, workplace bullying, turnover, distress and work  They also looked at anxiety and resilience | 1 study reported on Burnout (primary outcome) found significant lower levels of burnout compared to the control group – who had no intervention (P<.001)  Secondary outcomes were also reportedly improved | Cochrane’s Risk of Bias |
| Stratton et al. 2022 | Systematic Review and Meta-analysis | 2004 to July 2020 | 75 | Required to have a control group | Most studies included health care professionals (n = 18), insurance industry (n = 7), managers (n = 6), IT (n= 6) education (n=6), male-dominated industries (n = 5), telecommunications (n = 5), marketing and sales (n=3) banking (n=1) and HR (n=1) | Three most common interventions were based on CBT, Stress-management and mindfulness.  Platform used - Most interventions were delivered via web-based platforms as opposed to smartphone apps. | Depression, anxiety, stress | Found that the body of evidence for workplace digital interventions has tripled in the past decade, but no evidence to support effectiveness has increased.  Found small positive effects on anxiety  (Hedges g=0.26, 95% CI 0.13-0.39; P<.001)  For depression there was - small positive effects (Hedges g=0.26, 95% CI 0.19-0.34; P<.001)  And stress (Hedges g=0.25, 95% CI 0.17-0.34; P<.001) | Cochrane risk of bias tool for RCTs (RoB version 2.0) |
| Vertola et al. 2022 | Systematic Review | 2  January 2012 to January 2022 | 11 | Randomized controlled trials (RCTs) (10) and Clinical Trial (1) | Adult workers – sectors not specified | Mindfulness/meditation, Stress, Well-being, mental health psychoeducation, sleep quality, emotional regulation  Platform used - Mobile applications | Wellbeing - general and work-related  Anxiety, Depression, Stress, Perceived stress  Job Stress, Emotional Labor, Self-Regulation, Life satisfaction, Compassion satisfaction, Burnout | Range of outcomes, studies reported an Increase in wellbeing (n = 7), Reduction in perceived stress (n =4), Reduction in stress (n = 3), Decrease in anxiety symptoms (n = 2), Decrease in Depressive symptoms (n = 2), Reduction in burnout symptoms (n = 2) , Decrease in job stress (n = 2) | NR |
| Armaou et al. 2022 | Systematic Review | 5  January 1990 to July 2019 | 51 | RCT (n = 30) and Quasi-experimental research (n = 21) | RCTs were across multiple sectors, with the most in Healthcare (n = 8), technology/IT companies (n = 5), manufacturing (n = 3) Quasi- Experimental studies were across multiple sectors, most within Healthcare (n = 11), governmental or public enterprises (n = 3) and university employees (n = 2) | Categorised into four clusters of interventions, “self-help interventions” (n = 18), followed by Stress-management (n=14), Mindfulness/meditation (n=14) and CBT (n = 5)  Platform used - A mix of web-based and smart-phone based interventions | Primary Outcomes - Grouped into 3 areas:  Mental Health concerns: Depression, anxiety and dysfunctional attitudes. Work-related wellbeing: Perceived stress, psychological distress and job strain Psychological wellness: general mental wellbeing/positive mental health, happiness and life satisfaction. Mindfulness and resilience, self-efficacy, coping and gratitude. | Mental Health concerns: 10/51 (19.6%) Studies reported positive effects  Work-related wellbeing: 28/51 (54.9%) Studies reported positive effects  Psychological wellness indicators: 19/51 (37.3%) studies reported positive effects | Cochrane Collaboration’s Risk of Bias and JBI Critical Appraisal Checklist |
| Moe-Byrne 2022 | Systematic Review | 4  2000 onwards | 7 | RCTs, including one crossover RCT and two cluster RCTs | Participants were recruited from a variety of workplaces. Office based organisations (n=4) healthcare professionals (n=2) private and public sector (n = 1) | All studies used CBT as a theoretical background - 2 stated the use of mindfulness. Two used stress model or the job demands resources model -  Increase wellbeing - preventative interventions (n = 3), Work performance and Occupational health guidance (n = 1)  Platform used - A web-based intervention (n =5) a smartphone app intervention (n = 1) and a combined web based and smartphone app intervention (n = 1) | depression, anxiety and stress and work-related outcomes (absenteeism, presenteeism),  Physical measures - sleeping problems (n = 2), sleep and workplace performance (n=1),  somatisation (n = 1), physical health impairment (n = 1) | All studies reported psychological outcomes:  significant improvement for both anxiety and depression (n = 2)  significantly lower stress scores (n = 3)  Wellbeing – mixed results, significantly more wellbeing over time (n = 1) did not find a statistically significant positive effect(n = 1)  Other mental health outcomes: significant effects for positive mental health (F = 3.46, p = 0.03, Cohen’s d = 0.37 at three months follow-up, 0.28 at six months follow-up) (n = 2)  employee’s worry and quality of life regarding mental health (p <0.001 at six months (n = 1) | Cochrane Risk of Bias tool |
| Drissi et al. 2021 | Systematic Review | 5  2020 onwards | 11 | Mixed, only 3 included empirical evaluation of the interventions | Health care workers | Peer support (n = 2) E-learning (n = 3) online resources (n = 3) PTSD Coach ( n = 1) Headspace (n = 1) hotline (n = 1) screening (n = 1) Virtual care (n = 1)  Platform used - Social media (n = 2) Online support platform/resources (n =8) Mobile apps (n = 1) | Only 3 studies included an evaluation, 71% participants stated one platform helped them adjust faster to the situation, and another platform was used by 82% of participants in their work or home lives, another handbook was reported to have positive qualitative feedback | Lack of empirical evidence for health care workers, evidence mainly targeted health care workers in China | NR |
| Paganin and Simbula 2020 | Systematic Review | 2  2009 to 2019 | 31 | Quantitative or qualitative research design:  15 studies had control group, n = 10 were RCTs  6 Research Protocol (RP) studies, including 5 RCTs | General workers (n = 11), Health and social care (n = 8), Office-based (n = 5), Technology (n = 2), Middle managers (n = 1), Construction (n=1), Airplane pilots (n=1), Faculty members (n = 1), General workers with serious mental illness (n = 1) | Behavioural Change Techniques (n = 5)  Mindfulness (n = 3)  Stress models (n=3)  CBT (n = 2)  Other interventions/models such as Acceptance and Commitment Therapy  Did not report on theory used (n=12)  Platform used - Smart-phone based interventions | Stress-management, psychological wellbeing, secondary outcomes of resilience, and burnout | Studies reported on positive results for wellbeing and stress-management – on intervention effectiveness, usability, and feasibility | NR |
| Philips et al. 2019 | Systematic Review and Meta-analysis | 5  Database inception to April 2018 | 50 | RCTs | Varied sectors –  IT ( n= 7)  Healthcare (n = 6)  Education (n =3)  Communication and media (n = 3)  Public sector (n = 3)  Banking (2) | CBT (n = 22)  Personalised feedback – general health check (n = 7)  Mindfulness (n=6)  Psychoeducation (n = 5)  Remaining studies used a variety of training methods, such as cognitive, positive psychology or problem-solving  Platform used –  Web-based interventions (n = 47) smartphone- or app-based interventions (n = 3) | Stress Depression  Anxiety Burnout Insomnia  Mental wellbeing  mindfulness Alcohol Intake | 22 studies on stress had a medium positive effect on perceived stress (with g=0.54 (95% CI 0.35‒0.72, P<0.0001)  17 studies with depression as an outcome observed a significant small positive effect (g=0.30, 95% CI 0.18‒0.42, P<0.0001)  And 15 studies on anxiety had a small positive effect on anxiety (g=0.34; 95% CI 0.18‒0.50, P=0.0001). | Cochrane risk of-bias tool (RoB 2) |
| Howarth et al. 2018 | Systematic Review | 5  Database inception to December 2017 | 22 | RCTs | Varity of workplaces – public and private companies, healthcare professionals, education and manufacturing plants. | Interventions aimed at improving:  alcohol (n =5), mental health (n=5), sedentary behaviour (n = 3), musculoskeletal symptoms (n = 2), heart health (n = 2), insomnia (n = 1), mix of work-related rumination, fatigue and sleep (n = 1)  mix of outcomes including coping, diet, stress and general health (n = 3)  Platform used - Web-based (n = 11)  Web-based with Email (n = 5)  Web-based with both Email and SMS (n = 2)  Downloaded software (n = 2)  Web-based with SMS (n = 1)  Smartphone with SMS (n= 1) | Psychological measures - anxiety and depression (n = 6). Others include mindfulness and help-seeking attitude  Workplace measures - Job stress, work engagement, work productivity | Studies reported positive significant (n = 9) findings for:  sedentary behaviour (n = 3), mental health (n = 2), job satisfaction (n = 1)  diet, exercise, self-efficacy (n = 1) and insomnia (n = 1)  work-related levels of rumination, problem solving, pondering, fatigue and sleep quality (n = 1) | Cochrane’s Risk of Bias |
| Stratton et al.2017 | Systematic Review and Meta-analysis | 3  1975 to 17 November 2016 | 23 | RCT (n = 22) Controlled Trial (n = 1) | Studies were across multiple sectors - Including Education, Healthcare, Manufacturing, IT and Media | CBT intervention (n=11) Stress management (n = 6) Mindfulness-based approaches (n=6)  Platform used - Mixed - Web-based (n = 20) Smart-phone - (n =3) | Effectiveness – Stress, Anxiety, Depression | Overall, post intervention found significant small effect -  (g = 0.24, 95% CI 0.13 to 0.35, p = <0.00).  For CBT, a significant but very small positive effect was found  Mindfulness had a moderate to large effect, but stress management interventions produced a non-significant small positive effect | Downs and Black checklist  Risk of Bias using the Cochrane Guidelines |
| Carolan et al. 2017 | Systematic Review and Meta-analysis | 5 January 2000 to May 2016 | 21 | RCTs | Most studies were from the general working population (n = 4), and local authorities (n = 3), education (n = 3), technology (n = 2) | CBT - Based (n = 12), stress and coping (3) mindfulness (n = 2) social cognitive theory (n = 1), positive psychology (n = 1), problem solving training (n = 1), acceptance and commitment therapy (n = 1)  Self-guided (n=11) some guidance (n = 10)  Platform used - web-based (n = 17), computer application (2), email (n=1), standalone computer (n = 1) | Psychological  Well-being and work effectiveness | Found digital interventions had statistically significant positive effects on psychological well-being (g=0.37, 95% CI 0.23-0.50) and work effectiveness (g=0.25, 95% CI 0.09-0.41) when compared to the control group | Cochrane Collaboration’s risk of bias tool |
| Narvaez et al. 2014 | Systematic Review | 5  2003 - 2014 | 21 | Methodology needed to be included in the study | NR | CBT (n = 10) Combination of therapies (n =5)Problem solving therapy (n=1) other types of therapies (n = 5)  Platform used - web-based (n = 17), sensor networks (n=2), mobile (n=1) | Occupational stress | 12 studies had a positive effect on occupational stress, 3 had a positive effect but there not statistically significant, 2 studies had an indefinite effect | NR |
| López-Del-Hoyo et al. 2023 | Systematic Review | 3  Did not establish a date range. | 27 | Quantitative studies, including randomised  controlled trials (RCTs), nonrandomised trials, and single-arm studies | Healthcare workers | 22 interventions emerged, the authors classified digital interventions into their format - self-guided vs guided, and their contents - 'third-wave' pyschotherapies which described mindfulness interventions vs others  18/22 interventions were self-guided 14 of which were web-based, 3 smartphone apps and one based on text messages | Primary outcome - stress  depressive symptoms, anxiety, burnout, resilience and mindfulness | 13 interventions produced significant post treatment reductions in stress levels - there were also significant improvements found for depressive symptoms, anxiety, burnout, resilience and mindfulness. | Heart, Lung, and Blood  Institute assessment tools |

**References**

Drissi N, Ouhbi S, Marques G, de la Torre Díez I, Ghogho M, Janati Idrissi MA. A systematic literature review on e-Mental health solutions to assist health care workers during COVID-19. Telemed J E Health 2021; 27(6):594-602

Narváez S, Tobar AM, López DM. Systematic review of interventions supported by ICT for the prevention treatment of occupational stress. Stud Health Technol Inform 2014; 200:71-80

Paganin G, Simbula S. Smartphone-based interventions for employees' well-being promotion: a systematic review. Electron J Appl Stat Anal 2020; 13(03):682-712

Phillips EA, Gordeev VS, Schreyögg J. Effectiveness of occupational e-mental health interventions: a systematic review and meta-analysis of randomized controlled trials. Scand J Work Environ Health 2019; 45(6):560-576

Stratton E, Lampit A, Choi I, Calvo RA, Harvey SB, Glozier N. Effectiveness of eHealth interventions for reducing mental health conditions in employees: a systematic review and meta-analysis. PLoS One 2017; 12(12):e0189904

Vertola G, Marcello A, Bottone M, Sperandeo R, Muzii B, Scandurra C, Maldonato NM. Use and effectiveness of mobile health applications for stress management and emotional self-regulation in adult workers: a systematic review. 2022. Presented at: IEEE International Conference on Cognitive Infocommunications (CogInfoCom); 2022 September 23; Budapest, Hungary. p. 00081-00008

Moe-Byrne T, Shepherd J, Merecz-Kot D, Sinokki M, Naumanen P, Hakkaart-van Roijen L, Van Der Feltz-Cornelis C. Effectiveness of tailored digital health interventions for mental health at the workplace: a systematic review of randomised controlled trials. PLOS Digit Health 2022; 1(10):e0000123

Armaou M, Araviaki E, Dutta S, Konstantinidis S, Blake H. Effectiveness of digital interventions for deficit-oriented and asset-oriented psychological outcomes in the workplace: a systematic review and narrative synthesis. Eur J Investig Health Psychol Educ 2022; 12(10):1471-1497

Carolan S, Harris PR, Cavanagh K. Improving employee well-being and effectiveness: systematic review and meta-analysis of web-based psychological interventions delivered in the workplace. J Med Internet Res 2017; 19(7):e271

Howarth A, Quesada J, Silva J, Judycki S, Mills PR. The impact of digital health interventions on health-related outcomes in the workplace: a systematic review. Digit Health 2018; 4:2055207618770861

Park JH, Jung SE, Ha DJ, Lee B, Kim MS, Sim KL, Choi YH, Kwon CY. The effectiveness of e-healthcare interventions for mental health of nurses: a PRISMA-compliant systematic review of randomized controlled trials. Medicine (Baltimore) 2022; 101(25):e29125

Stratton E, Lampit A, Choi I, Malmberg Gavelin H, Aji M, Taylor J, Calvo RA, Harvey SB, Glozier N. Trends in effectiveness of organizational eHealth interventions in addressing employee mental health: systematic review and meta-analysis. J Med Internet Res 2022; 24(9):e37776

Xiong J, Wen JL, Pei GS, Han X, He DQ. Effectiveness of internet-based cognitive behavioural therapy for employees with depression: a systematic review and meta-analysis. Int J Occup Saf Ergon 2023; 29(1):268-281

López-Del-Hoyo Y, Fernández-Martínez S, Pérez-Aranda A, Barceló-Soler A, Bani M, Russo S, Urcola-Pardo F, Strepparava MG, García-Campayo J. Effects of eHealth interventions on stress reduction and mental health promotion in healthcare professionals: a systematic review. J Clin Nurs 2023; 32(17-18):5514-5533
