# Supplementary material for: Effectiveness of Digital Mental Health Interventions in the Workplace: Umbrella Review of Systematic Reviews
Source: JMIR Ment Health. 2025 Jan 24;12:e67785. doi: 10.2196/67785 (PMC11806266; doi:10.2196/67785)
Supplement: Multimedia Appendix 4 [file mental_v12i1e67785_app4.docx]

AMSTAR-2 Assessment Results
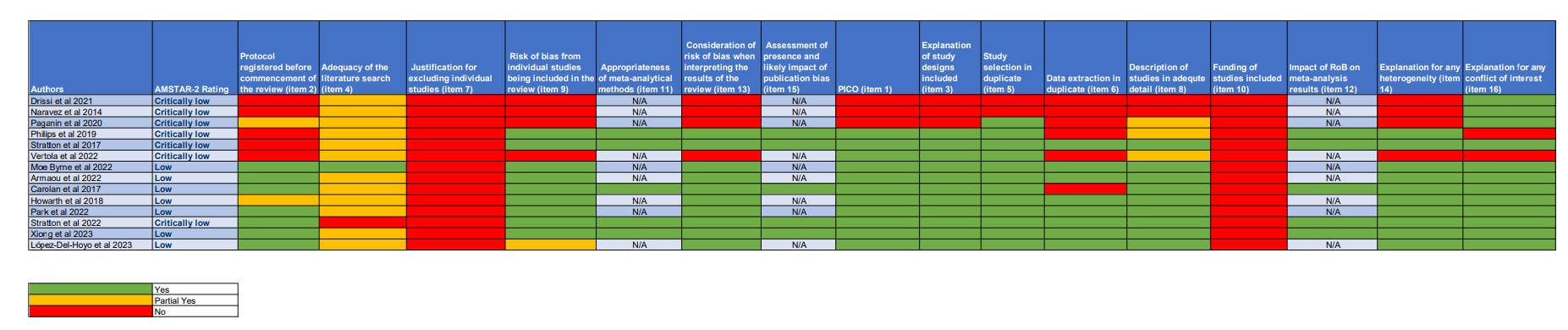


References:

Drissi N, Ouhbi S, Marques G, de la Torre Díez I, Ghogho M, Janati Idrissi MA. A systematic literature review on e-Mental health solutions to assist health care workers during COVID-19. Telemed J E Health 2021; 27(6):594-602

Narváez S, Tobar AM, López DM. Systematic review of interventions supported by ICT for the prevention treatment of occupational stress. Stud Health Technol Inform 2014; 200:71-80

Paganin G, Simbula S. Smartphone-based interventions for employees' well-being promotion: a systematic review. Electron J Appl Stat Anal 2020; 13(03):682-712

Phillips EA, Gordeev VS, Schreyögg J. Effectiveness of occupational e-mental health interventions: a systematic review and meta-analysis of randomized controlled trials. Scand J Work Environ Health 2019; 45(6):560-576

Stratton E, Lampit A, Choi I, Calvo RA, Harvey SB, Glozier N. Effectiveness of eHealth interventions for reducing mental health conditions in employees: a systematic review and meta-analysis. PLoS One 2017; 12(12):e0189904

Vertola G, Marcello A, Bottone M, Sperandeo R, Muzii B, Scandurra C, Maldonato NM. Use and effectiveness of mobile health applications for stress management and emotional self-regulation in adult workers: a systematic review. 2022. Presented at: IEEE International Conference on Cognitive Infocommunications (CogInfoCom); 2022 September 23; Budapest, Hungary. p. 00081-00008

Moe-Byrne T, Shepherd J, Merecz-Kot D, Sinokki M, Naumanen P, Hakkaart-van Roijen L, Van Der Feltz-Cornelis C. Effectiveness of tailored digital health interventions for mental health at the workplace: a systematic review of randomised controlled trials. PLOS Digit Health 2022; 1(10):e0000123

Armaou M, Araviaki E, Dutta S, Konstantinidis S, Blake H. Effectiveness of digital interventions for deficit-oriented and asset-oriented psychological outcomes in the workplace: a systematic review and narrative synthesis. Eur J Investig Health Psychol Educ 2022; 12(10):1471-1497

Carolan S, Harris PR, Cavanagh K. Improving employee well-being and effectiveness: systematic review and meta-analysis of web-based psychological interventions delivered in the workplace. J Med Internet Res 2017; 19(7):e271

Howarth A, Quesada J, Silva J, Judycki S, Mills PR. The impact of digital health interventions on health-related outcomes in the workplace: a systematic review. Digit Health 2018; 4:2055207618770861

Park JH, Jung SE, Ha DJ, Lee B, Kim MS, Sim KL, Choi YH, Kwon CY. The effectiveness of e-healthcare interventions for mental health of nurses: a PRISMA-compliant systematic review of randomized controlled trials. Medicine (Baltimore) 2022; 101(25):e29125

Stratton E, Lampit A, Choi I, Malmberg Gavelin H, Aji M, Taylor J, Calvo RA, Harvey SB, Glozier N. Trends in effectiveness of organizational eHealth interventions in addressing employee mental health: systematic review and meta-analysis. J Med Internet Res 2022; 24(9):e37776

Xiong J, Wen JL, Pei GS, Han X, He DQ. Effectiveness of internet-based cognitive behavioural therapy for employees with depression: a systematic review and meta-analysis. Int J Occup Saf Ergon 2023; 29(1):268-281

López-Del-Hoyo Y, Fernández-Martínez S, Pérez-Aranda A, Barceló-Soler A, Bani M, Russo S, Urcola-Pardo F, Strepparava MG, García-Campayo J. Effects of eHealth interventions on stress reduction and mental health promotion in healthcare professionals: a systematic review. J Clin Nurs 2023; 32(17-18):5514-5533
